# Common fixed point results on an extended b-metric space

**DOI:** 10.1186/s13660-018-1745-4

**Published:** 2018-07-03

**Authors:** Badr Alqahtani, Andreea Fulga, Erdal Karapınar

**Affiliations:** 1 Department of Mathematics, King Saud University, Riyadh, Saudi Arabia; 2 Department of Mathematics and Computer Sciences, Universitatea Transilvania Brasov, Brasov, Romania; 3 Department of Mathematics, Atilim University, Ankara, Turkey

**Keywords:** 46T99, 47H10, 54H25, Fixed point, Metric space

## Abstract

In this paper, we investigate the existence of common fixed points of a certain mapping in the frame of an extended b-metric space. The given results cover a number of well-known fixed point theorems in the literature. We state some examples to illustrate our results.

## Introduction and preliminaries

Throughout the manuscript, we denote $\mathbb{N}_{0}:=\mathbb{N}\cup\{0\} $, where $\mathbb{N}$ is the positive integers. Further, $\mathbb{R}$ represents the real numbers and $\mathbb{R}_{0}^{+}:=[0,\infty)$.

Following this pioneering result on *b*-metric, a number of authors have reported several interesting results in this direction (see, e.g., [1, 2, 4–12, 14, 16, 18] and the related references therein).

### Definition 1.1

(Czerwik [11])

Let *X* be a nonempty set and $d:X\times X\to[0,\infty)$ be a function satisfying the following conditions:

$(b1)$
$d(x,y)=0$ if and only if $x=y$.
$(b2)$
$d(x,y)=d(y,x)$ for all $x,y \in X$.
$(b3)$
$d(x,y)\leq s[d(x,z)+d(z,y)]$ for all $x,y,z\in X$, where $s\geq1$.

The function *d* is called a *b*-metric and the space $(X,d)$ is called a *b*-metric space, in short, bMS.

The immediate example of *b*-metric is the following.

### Example 1.1

Let $Y= \{ x,y,z \}$ and $X=Y\cup\mathbb {N} $. Define a mapping $d:X\times X\rightarrow[0,\infty)$ such that

$$\begin{aligned} &d ( x,y ) =d ( y,x ) =d ( x,z ) =d ( z,x ) =1, \\ &d ( y,z ) =d ( z,y ) =A, \\ &d (x,x ) =d ( y,y ) =d (z,z ) =0,\qquad d (n,m ) = \biggl\vert \frac{1}{n}- \frac{1}{m} \biggr\vert , \end{aligned}$$

where $A \in[2,\infty)$. Then we find that

$$ d ( x,y ) \leq\frac{A }{2} \bigl[ d ( x,z ) +d ( z,y ) \bigr] \quad\text{for }x,y,z \in X. $$

It is evident that $( X,d ) $ is a *b*-metric space. Notice also that if $A >2$, the standard triangle inequality does not hold and $( X,d ) $ is not a metric space.

### Remark 1.1

It is clear that for $s=1$, the *b*-metric becomes a usual metric.

Recently, Kamran [13] introduced a new type of generalized metric space and they proved some fixed point theorems on this space.

### Definition 1.2

([13])

Let *X* be a nonempty set and $\theta:X\times X\rightarrow[1,\infty)$. A function $d_{\theta}:X\times X\rightarrow[0,\infty)$ is called an extended *b*-metric if, for all $x,y,z\in X$, it satisfies

$(d_{\theta}1)$
$d_{\theta}(x,y)=0 $ iff $x=y$;
$(d_{\theta}2)$
$d_{\theta}(x,y)=d_{\theta}(y,x)$;
$(d_{\theta}3)$
$d_{\theta}(x,y)\leq\theta(x,y) [d_{\theta}(x,z)+d_{\theta}(z,y) ] $.

The pair $(X,d_{\theta})$ is called an extended *b*-metric space, in short extended-*b*MS.

### Remark 1.2

If $\theta(x, y)=s$ for $s\geq1$, then we obtain the definition of *b*MS.

### Example 1.2

Let $X=[0,1]$ and $\theta:X\times X\rightarrow[1,\infty)$, $\theta (x,y)=\frac{x+y+1}{x+y}$. Define $d_{\theta}:X\times X\rightarrow[0,\infty )$ as

$$\begin{aligned} &d_{\theta}(x, y)=\frac{1}{xy} \quad\text{for }x,y\in(0,1], x\neq y, \\ &d_{\theta}(x,y)=0 \quad\text{for }x,y\in[0,1], x=y, \\ &d_{\theta}(x,0)=d_{\theta}(0,x)=\frac{1}{x} \quad\text{for }x \in(0,1]. \end{aligned}$$

Obviously, $(d_{\theta}1)$ and $(d_{\theta}2)$ hold. For $(d_{\theta}3)$, we distinguish the following cases:

(i) Let $x,y\in(0,1]$. For $z\in(0,1]$, we have
  $$d_{\theta}(x, y)\leq\theta(x,y)\bigl[d_{\theta}(x,z)+d_{\theta}(z,y) \bigr] \quad\Leftrightarrow\quad\frac{1}{xy}\leq\frac{1+x+y}{x+y}\cdot \frac{x+y}{xyz} \quad\Leftrightarrow\quad z\leq1+x+y. $$
  If $z=0$, then
  $$d_{\theta}(x, y)\leq\theta(x,y)\bigl[d_{\theta}(x,0)+d_{\theta}(0,y) \bigr] \quad\Leftrightarrow\quad\frac{1}{xy}\leq\frac{1+x+y}{x+y}\cdot \frac{x+y}{xy} \quad\Leftrightarrow\quad 1\leq1+x+y. $$
(ii) For $x\in(0,1]$ and $y=0$, let $z\in(0,1]$.
  $$d_{\theta}(x, 0)\leq\theta(x,0)\bigl[d_{\theta}(x,z)+d_{\theta}(z,0) \bigr] \quad\Leftrightarrow\quad\frac{1}{x}\leq\frac{1+x}{x}\cdot \frac{1+x}{xz} \quad\Leftrightarrow\quad xz\leq(1+x)^{2}. $$
  In conclusion, for any $x, y, z\in X$,
  $$d_{\theta}(x,z)\leq\theta(x,z) \bigl[d_{\theta}(x,y)+d_{\theta}(y,z) \bigr]. $$
  Hence, $(X,d_{\theta})$ is an extended *b*-metric space.

Some fundamental concepts, like convergence, Cauchy sequence, and completeness in a extended-*b*MS, are defined as follows [13].

### Definition 1.3

([13])

Let $(X,d_{\theta})$ be an extended-*b*MS.

(i) A sequence ${x_{n}}$ in *X* is said to converge to $x\in X$ if, for every $\epsilon>0$, there exists $N=N(\epsilon)\in\mathbb{N}$ such that $d_{\theta}(x_{n}, x)<\epsilon$ for all $n\geq N$. In this case, we write $\lim_{n\rightarrow\infty} x_{n} = x$.
(ii) A sequence ${x_{n}}$ in *X* is said to be Cauchy if, for every $\epsilon>0$, there exists $N=N(\epsilon)\in\mathbb{N}$ such that $d_{\theta}(x_{m}, x_{n})<\epsilon$ for all $m,n\geq N$.

### Definition 1.4

An extended-*b*-metric space $(X,d_{\theta})$ is complete if every Cauchy sequence in *X* is convergent.

### Lemma 1.1

*Let* $(X,d_{\theta})$ *be a complete extended*-*bMS*. *If* $d_{\theta}$ *is continuous*, *then every convergent sequence has a unique limit*.

### Theorem 1.1

([13])

*Let* $(X,d_{\theta})$ *be an extended*-*bMS such that* $d_{\theta}$ *is a continuous functional*. *Let* $T:X\rightarrow X$ *satisfy*

1 $$ d_{\theta}(Tx,Ty)\leq k d_{\theta}(x,y) $$

*for all* $x,y\in X $, *where* $k\in [0,1 )$ *is such that*, *for each* $x_{0}\in X$, $\lim_{n,m\rightarrow\infty} \theta(x_{n}, x_{m})<\frac{1}{k}$, *here* $x_{n}=T^{n}x_{0}$, $n=1,2,\ldots $ . *Then* *T* *has precisely one fixed point* *u*. *Moreover*, *for each* $y\in X$, $T^{n}y\rightarrow u$.

For our purposes, we need to recall the following definition which is proposed by Popescu [17].

### Definition 1.5

Let $T:X\rightarrow X$ and $\alpha:X\times X\rightarrow [0, \infty )$. We say that *T* is an *α*-orbital admissible if, for all $x,y\in X$, we have

2 $$ \alpha(x, Tx)\geq1\quad \Rightarrow\quad\alpha\bigl(Tx, T^{2}x \bigr)\geq1. $$

### Definition 1.6

A set *X* is regular with respect to mapping $\alpha:X\times X \to [0,\infty)$ if, whenever $\{x_{n}\}$ is a sequence in *X* such that $\alpha (x_{n},x_{n+1})\geq1$ and $\alpha(x_{n+1},x_{n})\geq1$ for all *n* and $x_{n} \rightarrow x\in X$ as $n\rightarrow\infty$, then there exists a subsequence $\{x_{n(k)} \}$ of $\{ x_{n} \}$ such that $\alpha(x_{n(k)},x)\geq1$ and $\alpha(x,x_{n(k)})\geq1$ for all *n*.

## (S,T) orbital cyclic

### Definition 2.1

Suppose that $T,S$ are two self-mappings on a complete extended-*b*MS $(X, d_{\theta})$. Suppose also that there are two functions $\alpha,\beta :X\times X\rightarrow [0,\infty )$ such that, for any $x\in X$,

3 $$ \begin{aligned} &\alpha(x, Tx)\geq1 \quad\Rightarrow\quad\beta(Tx, STx)\geq1 \quad\text{and} \\ &\beta(x,Sx)\geq1\quad \Rightarrow\quad\alpha(Sx, TSx)\geq1. \end{aligned} $$

Then we say that the pair $S,T$ is an $(\alpha,\beta)$-orbital-cyclic admissible pair.

We start with the following lemma which is essential in our main results.

### Lemma 2.1

([3])

*Let* $(X,d_{\theta})$ *be an extended* *b*-*metric space*.*If there exists* $q\in [0,1 )$ *such that the sequence* $\{ x_{n} \}$ *for an arbitrary* $x_{0}\in X$ *satisfies* $\lim_{n,m\rightarrow\infty} \theta(x_{n}, x_{m})<\frac{1}{q}$, *and also*

4 $$ 0< d_{\theta}(x_{n}, x_{n+1})\leq q d_{\theta}(x_{n-1}, x_{n}) $$

*for any* $n\in\mathbb {N}$, *then the sequence* $\{x_{n}\}$ *is Cauchy in* *X*.

### Proof

Let $\{x_{n} \}_{n\in\mathbb {N}}$ be a given sequence. By employing inequality (4) recursively, we derive that

5 $$ 0< d_{\theta}(x_{n}, x_{n+1})\leq q^{n} d_{\theta}(x_{0}, x_{1}). $$

Since $q\in [0,1 )$, we find that

6 $$ \lim_{n\rightarrow\infty}d_{\theta}(x_{n}, x_{n+1})=0. $$

On the other hand, by $(d_{\theta}3)$, together with triangular inequality, for $p\geq1$, we derive that

7 $$\begin{aligned} & d_{\theta}(x_{n}, x_{n+p}) \\ &\quad\leq\theta(x_{n}, x_{n+p})\cdot \bigl[d_{\theta}(x_{n}, x_{n+1})+d_{\theta}(x_{n+1}, x_{n+p}) \bigr] \\ &\quad\leq\theta(x_{n}, x_{n+p}) d_{\theta}(x_{n}, x_{n+1})+\theta(x_{n}, x_{n+p})d_{\theta}(x_{n+1}, x_{n+p}) \\ &\quad\leq\theta(x_{n}, x_{n+p}) q^{n} d_{\theta}(x_{0}, x_{1})+\theta(x_{n}, x_{n+p})\theta(x_{n+1}, x_{n+p})\bigl[d_{\theta}(x_{n+1}, x_{n+2})+d_{\theta}(x_{n+2}, x_{n+p})\bigr] \\ & \quad\leq\theta(x_{n}, x_{n+p})\cdot q^{n} d_{\theta}(x_{0}, x_{1})+\theta(x_{n}, x_{n+p}) \theta(x_{n+1}, x_{n+p}) \cdot q^{n+1} d_{\theta}(x_{0}, x_{1})+\cdots \\ &\qquad{} +\theta(x_{n}, x_{n+p})\cdots\theta(x_{n+p-1}, x_{n+p})\cdot q^{n+p-1} d_{\theta}(x_{0}, x_{1}) \\ &\quad = d_{\theta}(x_{0}, x_{1}) \sum _{i=1}^{n+p-1} q^{i} \prod _{j=1}^{i} \theta (x_{n+j}, x_{n+p}). \end{aligned}$$

Notice the inequality above is dominated by $\sum_{i=1}^{n+p-1} q^{i} \prod_{j=1}^{i} \theta(x_{n+j}, x_{n+p}) \leq \sum_{i=1}^{n+p-1} q^{i}\times \prod_{j=1}^{i} \theta(x_{j}, x_{n+p}) $.

On the other hand, by employing the ratio test, we conclude that the series $\sum_{i=1}^{\infty}a_{i}$, where $a_{i}=q^{i} \prod_{j=1}^{i} \theta (x_{j}, x_{n+p})$ converges to some $S \in(0,\infty)$. Indeed, $\lim_{i\rightarrow\infty}\frac{a_{i+1}}{a_{i}} = \lim_{i\rightarrow\infty} q \theta(x_{i}, x_{i+p})<1$, and hence we get the desired result. Thus, we have

$$ S= \sum_{i=1}^{\infty}q^{i} \prod _{j=1}^{i} \theta(x_{j}, x_{n+p})\quad \text{with the partial sum } S_{n}= \sum _{i=1}^{n} q^{i} \prod _{j=1}^{i} \theta (x_{j}, x_{n+p}). $$

Consequently, we observe, for $n\leq1, p\leq1$, that

8 $$ d_{\theta}(x_{n}, x_{n+p})\leq q^{n} d_{\theta}(x_{0}, x_{1}) [S_{n+p-1}-S_{n-1}]. $$

Letting $n\rightarrow\infty$ in (8), we conclude that the constructive sequence $\{x_{n}\}$ is Cauchy in the extended *b*-metric space $(X, d_{\theta})$. □

### Theorem 2.1

*Let* $T,S$ *be two self*-*mappings on a complete extended*-*bMS* $(X,d_{\theta})$ *such that the pair* $T,S$ *forms an* $(\alpha,\beta)$-*orbital*-*cyclic admissible pair*. *Suppose that*

(i) *for each* $x_{0}\in X$, $\lim_{n,m\rightarrow\infty}\theta (x_{n},x_{m})<\frac{1-k}{k}$, *where* $x_{2n}=Sx_{2n-1}\textit{ and }x_{2n+1}=Tx_{2n}$ *for each* $n \in\mathbb {N}$;
(ii) *there exists* $x_{0}\in X$ *such that* $\alpha(x_{0},Tx_{0})\geq1$;
(iii) *either* *S* *and* *T* *are continuous*, *or*
(iii*) *if* ${x_{n}}$ *is a sequence in* *X* *such that* $x_{n}\rightarrow u$, *then* $\alpha(u, Tu)\geq1$ *and* $\beta(u,Su)\geq1$.

*Moreover*, *if for all* $x,y\in X$ *and* $k\in [0,\frac{1}{2} )$

9 $$ \alpha(x,Tx)\beta(y,Sy)d_{\theta}(Tx,Sy)\leq k \bigl[d_{\theta}(x,Tx)+d_{\theta}(y,Sy) \bigr], $$

*then the pair of the mappings* $T,S$ *possesses a common fixed point* *u*, *that is*, $Tu=u=Su$.

### Proof

By assumption (ii), there exists a point $x_{0}\in X$ such that $\alpha (x_{0},Tx_{0})\geq1$. Take $x_{1}=Tx_{0}$ and $x_{2}=Sx_{1}$. By induction, we construct a sequence $\{x_{n}\}$ such that

10 $$ x_{2n}=Sx_{2n-1}\quad \text{and}\quad x_{2n+1}=Tx_{2n} \quad\forall n \in\mathbb{N}. $$

We have $\alpha(x_{0},x_{1})\geq1$, and since $(S,T)$ is an $\alpha,\beta $-orbital-cyclic admissible pair, we get

$$ \alpha(x_{0},x_{1})\geq1 \quad\Rightarrow\quad\beta(Tx_{0}, STx_{0})=\beta(x_{1}, x_{2})\geq1 $$

and

$$ \beta(x_{1},x_{2})\geq1 \quad\Rightarrow\quad\alpha(Sx_{1},TSx_{1})= \alpha(x_{2}, x_{3})\geq1. $$

Applying again (3),

$$ \alpha(x_{2},x_{3})\geq1 \quad\Rightarrow\quad\beta(Tx_{2},STx_{2})= \beta(x_{3}, x_{4})\geq1 $$

and

$$ \beta(x_{3},x_{4})\geq1 \quad\Rightarrow\quad\alpha(Sx_{3},TSx_{3})= \alpha(x_{4}, x_{5})\geq1. $$

Recursively, we obtain

11 $$ \alpha(x_{2n},x_{2n+1})\geq1\quad \text{for all } n \in\mathbb{N}, $$

and

12 $$ \beta(x_{2n+1},x_{2n+2})\geq1 \quad\text{for all } n \in\mathbb{N}. $$

Without loss of generality, we assume that $x_{n}\neq x_{n+1}$ for each $n\in\mathbb{N}_{0}$. Indeed, if $x_{n_{0}}=x_{n_{0}+1}$ for some $n_{0}\in \mathbb{N}_{0}$, then $u=x_{n_{0}}$ forms a common fixed point for *S* and *T*, which finalizes the proof. More precisely, to see that *u* is the common fixed point of *S* and *T*, we shall examine the following two cases. First, we assume that $n_{0}$ is even, that is, $n_{0}=2k$. In this case, we have $x_{2k}=x_{2k+1}=Tx_{2k}$, that is, $x_{2k}$ is a fixed point of *T*. Now we shall prove that $x_{2k}=x_{2k+1}=Tx_{2k}=Sx_{2k+1}$. Suppose on the contrary that $d_{\theta}(Tx_{2k}, Sx_{2k+1})>0$. By letting $x=x_{2k}$ and $y=x_{2k+1}$ in (9) and keeping in mind (11) and (12), we get that

$$\begin{aligned} 0< d_{\theta}(x_{2k+1}, x_{2k+2})&= d_{\theta}(Tx_{2k}, Sx_{2k+1})\leq \alpha(x_{2k},Tx_{2k})\beta(x_{2k+1},Sx_{2k+1})d_{\theta}(Tx_{2k}, Sx_{2k+1}) \\ & \leq k \bigl[d_{\theta}(x_{2k},Tx_{2k})+d_{\theta}(x_{2k+1},Sx_{2k+1}) \bigr] \\ & \leq k d_{\theta}(x_{2k+1},Sx_{2k+1})=kd_{\theta}(x_{2k+1},x_{2k+2}), \end{aligned}$$

a contradiction. Hence, we conclude that $d_{\theta}(Tx_{2k}, Sx_{2k+1})=0$ and $x_{2k}=x_{2k+1}=Tx_{2k}=Sx_{2k+1}$, that is, $x_{2k}=x_{2k+1}=u$ is a common fixed point of *T* and *S*. Analogously, one can derive the same conclusion for the case $n_{0}$ is odd, that is, $n_{0}=2k-1$.

Thus, throughout the proof, we suppose that

13 $$ x_{n}\neq x_{n+1} \quad\text{for all } n\in \mathbb{N}_{0}. $$

In what follows, we shall prove that the sequence $\{x_{n}\}$ is Cauchy. For this purpose, it is sufficient to examine the following two cases.

Case (a): Let $x=x_{2n}$ and $y=x_{2n+1}$. Then, by inequality (9) and using (11), (12), we get

14 $$\begin{aligned} 0&< d_{\theta}(x_{2n+1}, x_{2n+2}) \\ &= d_{\theta}(Tx_{2n}, Sx_{2n+1})\leq \alpha(x_{2n},Tx_{2n})\beta(x_{2n+1},Sx_{2n+1})d_{\theta}(Tx_{2n}, Sx_{2n+1}) \\ & \leq k \bigl[d_{\theta}(x_{2n},Tx_{2n})+d_{\theta}(x_{2n+1},Sx_{2n+1}) \bigr] \\ & = k \bigl[d_{\theta}(x_{2n},x_{2n+1})+d_{\theta}(x_{2n+1},x_{2n+2}) \bigr], \end{aligned}$$

and from here

15 $$ d_{\theta}(x_{2n+1}, x_{2n+2})\leq q d_{\theta}(x_{2n}, x_{2n+1}) $$

for each $n\in\mathbb{N}_{0}$, where $q=\frac{k}{1-k}<1$ with $k\in [0, \frac{1}{2} )$.

Case (b): Let $x=x_{2n}$ and $y=x_{2n-1}$. Then, by inequality (9) and using (11), (12), we get

16 $$\begin{aligned} 0&< d_{\theta}(x_{2n+1}, x_{2n}) \\ &= d_{\theta}(Tx_{2n}, Sx_{2n-1})\leq \alpha (x_{2n},Tx_{2n})\beta(x_{2n-1},Sx_{2n-1})d_{\theta}(Tx_{2n}, Sx_{2n-1}) \\ & \leq k \bigl[d_{\theta}(x_{2n},Tx_{2n})+d_{\theta}(x_{2n-1},Sx_{2n-1}) \bigr] \\ & = k \bigl[d_{\theta}(x_{2n},x_{2n+1})+d_{\theta}(x_{2n-1},x_{2n}) \bigr] \end{aligned}$$

and

17 $$ d_{\theta}(x_{2n}, x_{2n+1})\leq q d_{\theta}(x_{2n-1}, x_{2n}) $$

for each $n\in\mathbb{N}_{0}$, where $q=\frac{k}{1-k}<1$ with $k\in [0, \frac{1}{2} )$.

Combining (15) and (17), we can conclude that

18 $$ d_{\theta}(x_{m}, x_{m+1})\leq q d_{\theta}(x_{m-1}, x_{m}) $$

for all $m\in\mathbb{N}$. From Lemma 2.1, taking into account (i), $\lim_{n,m\rightarrow \infty}\theta(x_{n},x_{m})<\frac{1-k}{k}=\frac{1}{q}$, we obtain that $\{x_{m} \}$ is a Cauchy sequence. By completeness of $(X,d_{\theta})$, there is some point $u\in X$ such that

19 $$ \lim_{n\rightarrow\infty}x_{n}=u. $$

Naturally, we also have

20 $$ x_{2n}\rightarrow u \quad\text{and}\quad x_{2n+1} \rightarrow u. $$

Due to the continuity of the mappings *T* and *S*, we get

$$u= \lim_{n\rightarrow\infty}x_{n+1}= \lim_{n\rightarrow\infty}Tx_{n}=T \lim_{n\rightarrow\infty}x_{n}=Tu $$

and

$$u= \lim_{n\rightarrow\infty}x_{n+1}= \lim_{n\rightarrow\infty}Sx_{n}=S \lim_{n\rightarrow\infty}x_{n}=Su. $$

Let us consider now the alternative hypothesis (iii*). Taking $x=u$ and $y=x_{2n+1}$ in (9) and taking into account (12), we get

21 $$\begin{aligned} d_{\theta}(Tu, x_{2n+2})&=d_{\theta}(Tu, Tx_{2n+1}) \\ & \leq\alpha(u,Tu)\beta (x_{2n+1},Sx_{2n+1})d_{\theta}(Tu,Sx_{2n+1}) \\ &\leq k \bigl[d_{\theta}(u,Tu)+d_{\theta}(x_{2n+1},x_{2n+2}) \bigr]. \end{aligned}$$

Letting $n\rightarrow\infty$, we obtain

22 $$\begin{aligned} d_{\theta}(Tu,u)&=\lim_{n\rightarrow\infty}d_{\theta}(Tu, x_{2n+2})\leq k\lim_{n\rightarrow\infty} \bigl[d_{\theta}(u,Tu)+d_{\theta}(x_{2n+1},x_{2n+2}) \bigr] \\ &=kd_{\theta}(u,Tu)< d_{\theta}(u,Tu), \end{aligned}$$

which implies $d_{\theta}(Tu,u)=0$. Hence, we get that $Tu=u$. Analogously, regarding (11) and (22), we observe that

23 $$\begin{aligned} d_{\theta}(x_{2n+1}, Su)&=d_{\theta}(Tx_{2n}, Su) \\ & \leq\alpha (x_{2n},Tx_{2n}) \beta(u,Su)d_{\theta}(Tx_{2n}, Su) \\ &\leq k \bigl[d_{\theta}(x_{2n},Tx_{2n})+d_{\theta}(u, Su) \bigr]. \end{aligned}$$

Now, letting $n\rightarrow\infty$ in the inequality above, we derive that

24 $$\begin{aligned} d_{\theta}(u,Su)&=\lim_{n\rightarrow\infty}d_{\theta}(x_{2n+1}, Su)\leq k\lim {n\rightarrow\infty} \bigl[d_{\theta}(x_{2n},Tx_{2n})+d_{\theta}(u,Su) \bigr] \\ &=kd_{\theta}(u,Su)< d_{\theta}(u,Su). \end{aligned}$$

Hence, we find that $Su=u$. Accordingly, we conclude that *T* and *S* have a common fixed point *u*. □

### Example 2.1

Let $X=[0,1]$ and $d_{\theta}:X\times X\rightarrow [0,\infty )$ defined by

$$\begin{aligned} &d_{\theta}(x, y)=\frac{1}{xy} \quad\text{for }x,y\in(0,1], x\neq y, \\ &d_{\theta}(x,y)=0 \quad\text{for }x,y\in[0,1], x=y, \\ &d_{\theta}(x,0)=d_{\theta}(0,x)=\frac{1}{x} \quad\text{for }x \in(0,1] \end{aligned}$$

when

$$ \theta(x,y)= \textstyle\begin{cases} \frac{1+x+y}{x+y} & \text{if }x \in (0,1 ],\\ 1 & \text{if }x=y=0. \end{cases} $$

Then $(X, d_{\theta})$ is an extended-*b*MS (see Example 1.2).

Let $T:X\rightarrow X$, $S:X\rightarrow X$, defined as

$$T(x)= \textstyle\begin{cases} 1 & \text{if }x=\frac{1}{2},\\ \frac{1}{2} & \text{if }x=\frac{1}{4},\\ \frac{x+1}{2} & \text{otherwise}, \end{cases} $$

respectively

$$S(x)=\textstyle\begin{cases} 1 & \text{if }x\in \{\frac{1}{2}, \frac{1}{4} \},\\ x & \text{otherwise}, \end{cases} $$

and two functions $\alpha, \beta:X\times X\rightarrow [0,\infty )$ defined by

$$\alpha(x,y)=\textstyle\begin{cases} 1 & \text{if } (x,y)\in \{ (1,1 ), (\frac {1}{2},1 ), (\frac{1}{4}, \frac{1}{2} ) \},\\ 0 & \text{otherwise}, \end{cases} $$

and

$$\beta(x,y)=\textstyle\begin{cases} 1 & \text{if } (x,y)\in \{ (1,1 ), (\frac {1}{2},1 ), (\frac{1}{4}, 1 ) \},\\ 0 & \text{otherwise}. \end{cases} $$

We show that the pair $T,S$ forms an $(\alpha,\beta)$-orbital-cyclic admissible pair. Indeed, for $x=1$,

$$\alpha(1, T1)=\alpha(1,1)\geq1 \quad\Rightarrow\quad \beta(T1, ST1)=\beta(1,1)\geq1 $$

and

$$\beta(1, S1)=\beta(1,1)\geq1 \quad\Rightarrow\quad\alpha(S1, TS1)=\alpha(1,1)\geq1. $$

For $x=\frac{1}{2}$:

$$\alpha \biggl(\frac{1}{2}, T\frac{1}{2} \biggr)=\alpha \biggl( \frac {1}{2},1 \biggr)\geq1 \quad\Rightarrow\quad\beta \biggl(T\frac{1}{2}, ST \frac {1}{2} \biggr)=\beta (1,1 )\geq1 $$

and

$$\beta \biggl(\frac{1}{2}, S\frac{1}{2} \biggr)=\beta \biggl( \frac {1}{2},1 \biggr)\geq1 \quad\Rightarrow\quad\alpha \biggl(S\frac{1}{2}, TS \frac {1}{2} \biggr)=\alpha (1,1 )\geq1. $$

For $x=\frac{1}{4}$:

$$\alpha \biggl(\frac{1}{4}, T\frac{1}{4} \biggr)=\alpha \biggl( \frac {1}{4},\frac{1}{2} \biggr)\geq1 \quad\Rightarrow\quad\beta \biggl(T \frac{1}{4}, ST\frac{1}{4} \biggr)=\beta \biggl(\frac{1}{2},1 \biggr)\geq1 $$

and

$$\beta \biggl(\frac{1}{4}, S\frac{1}{4} \biggr)=\beta \biggl( \frac {1}{4},1 \biggr)\geq1 \quad\Rightarrow\quad\alpha \biggl(S\frac{1}{4}, TS \frac {1}{4} \biggr)=\alpha (1,1 )\geq1. $$

We have thus proved that *T* is *α* orbital admissible, and sure, because $\alpha(\frac{1}{4},T\frac{1}{4})\geq1$, assumption (ii) is satisfied.

If $x_{0}\in \{\frac{1}{4}, \frac{1}{2}, 1 \}$, then $x_{n}=T^{n}x_{0}=1$, so

$$\lim_{n,m\rightarrow\infty}\theta(x_{n}, x_{m})= \frac{3}{2}< 3=\frac{1-k}{k}, $$

where we choose $k=\frac{1}{4}<\frac{1}{2}$. Otherwise, for each $x_{0}\in X - \{\frac{1}{4}, \frac{1}{2}, 1 \}$, we have $x_{2n-1}=\sum_{k=1}^{n} (\frac{1}{2} )^{n}+\frac{x_{0}}{2^{n}}$, $x_{2n}=x_{2n-1}$ and $\lim_{n\rightarrow\infty}x_{n}=1$. So,

$$\lim_{n,m\rightarrow\infty}\theta (x_{n}, x_{m} )= \frac {3}{2}< 3=\frac{1-k}{k}. $$

Hence, (i) is also verified.

We have

$$\begin{aligned} &d_{\theta}(1,T1)=0,\qquad d_{\theta}\biggl(\frac{1}{2}, T \frac{1}{2}\biggr)=2,\qquad d_{\theta}\biggl(\frac {1}{4}, T \frac{1}{4}\biggr)=8, \\ &d_{\theta}(1, S1)=0, \qquad d_{\theta}\biggl(\frac{1}{2}, S \frac{1}{2}\biggr)=2,\qquad d_{\theta}\biggl(\frac {1}{4}, S \frac{1}{4}\biggr)=4 \end{aligned}$$

and

$$\begin{aligned} &d_{\theta}(T1,S1)=0,\qquad d_{\theta}\biggl(T1,S\frac{1}{2} \biggr)=0, \qquad d_{\theta}\biggl(T1,S\frac{1}{4}\biggr)=0, \\ &d_{\theta}\biggl(T\frac{1}{2},S1\biggr)=0,\qquad d_{\theta}\biggl(T \frac{1}{2},S\frac{1}{2}\biggr)=0,\qquad d_{\theta}\biggl(T \frac{1}{2},S\frac{1}{4}\biggr)=0, \\ &d_{\theta}\biggl(T\frac{1}{4},S1\biggr)=2,\qquad d_{\theta}\biggl(T \frac{1}{4},S\frac{1}{2}\biggr)=2,\qquad d_{\theta}\biggl(T \frac{1}{4},S\frac{1}{4}\biggr)=2. \end{aligned}$$

Because in the other cases $\alpha(x,y)=0$ and $\beta(x,y)=0$, it is enough to investigate the following situations:

Case (a): For $x\in \{1,\frac{1}{2} \}$ and $y\in \{1, \frac{1}{2}, \frac{1}{4} \}$,

$$ d_{\theta}(Tx,Sy)=0 $$

so inequality (9) is satisfied.

Case (b): Let $x=\frac{1}{4}, y=1$. Then

$$\begin{aligned} 2&=d_{\theta}\biggl(T\frac{1}{4},S1\biggr)= \alpha\biggl( \frac{1}{4}, \frac{1}{2}\biggr)\beta (1,1)d_{\theta}\biggl(T \frac{1}{4},S1\biggr)\\ & \leq\frac{1}{4} \biggl[d_{\theta}\biggl( \frac {1}{4}, T\frac{1}{4}\biggr)+d_{\theta}(1, S1) \biggr]= \frac{1}{4} [8+0 ]=\frac{8}{4}. \end{aligned}$$

Case (c): Let $x=\frac{1}{4}, y=\frac{1}{2}$. Then

$$\begin{aligned} 2&=d_{\theta}\biggl(T\frac{1}{4},S\frac{1}{2}\biggr)=\alpha \biggl(\frac{1}{4}, T\frac {1}{4}\biggr)\beta\biggl( \frac{1}{2},S\frac{1}{2}\biggr)d_{\theta}\biggl(T \frac{1}{4},S\frac {1}{2}\biggr)\\ & \leq\frac{1}{4} \biggl[d_{\theta}\biggl(\frac{1}{4}, T\frac {1}{4} \biggr)+d_{\theta}\biggl(\frac{1}{2}, S\frac{1}{2}\biggr) \biggr]=\frac{1}{4} [8+2 ]=\frac{10}{4}. \end{aligned}$$

Case (d): Let $x=\frac{1}{4}, y=\frac{1}{4}$. Then

$$\begin{aligned} 2&= \alpha\biggl(\frac{1}{4}, T\frac{1}{4}\biggr)\beta\biggl( \frac{1}{4},S\frac {1}{4}\biggr)d_{\theta}\biggl(T \frac{1}{4},S\frac{1}{4}\biggr)\\ & \leq\frac{1}{4} \biggl[d_{\theta}\biggl(\frac{1}{4}, T\frac{1}{4} \biggr)+d_{\theta}(\frac{1}{4}, S\frac {1}{4} \biggr]= \frac{1}{4} [8+4 ]=\frac{12}{4}. \end{aligned}$$

Therefore, all the conditions of Theorem 2.2 are satisfied and *T* has a unique fixed point, $x=1$.

### $(\alpha,\beta)$-orbital-cyclic

#### Definition 2.2

Let *X* be a nonempty set, $T:X\rightarrow X$, and $\alpha,\beta :X\times X\rightarrow [0,\infty )$. We say that *T* is an $(\alpha,\beta)$-orbital-cyclic admissible mapping if

25 $$ \begin{aligned}&\alpha(x,Tx) \geq1 \quad\text{implies } \beta\bigl(Tx, T^{2}x \bigr)\geq1 \quad\text{and}\\ & \beta(x,Tx) \geq1 \quad\text{implies } \alpha\bigl(Tx, T^{2}x\bigr)\geq1 \end{aligned}$$

for all $x\in X$.

#### Corollary 2.1

*Let* *T* *be a self*-*mapping on a complete extended*-*bMS* $(X,d_{\theta})$ *such that the mapping* *T* *forms an* $(\alpha,\beta)$-*orbital*-*cyclic admissible mapping*. *Suppose that*

(i) *for each* $x_{0}\in X$, $\lim_{n,m\rightarrow\infty}\theta (x_{n},x_{m})<\frac{1-k}{k}$, *where* $x_{n}=T^{n}x_{0}$, $n=1,2,\ldots $ ;
(ii) *there exists* $x_{0}\in X$ *such that* $\alpha (x_{0},Tx_{0})\geq1$ *and* $\beta(x_{0}, Tx_{0})\geq1$;
(iii) *either* *T* *is continuous*, *or*
(iii*) *if* ${x_{n}}$ *is a sequence in* *X* *such that* $x_{n}\rightarrow u$, *then* $\alpha(u, Tu))\geq1$ *and* $\beta(u, Tu)\geq1$.

*Moreover*, *if for all* $x,y\in X$ *and* $k\in [0,\frac{1}{2} )$

26 $$ \alpha(x,Tx)\beta(y,Ty)d_{\theta}(Tx,Ty)\leq k \bigl[d_{\theta}(x,Tx)+d_{\theta}(y,Ty) \bigr], $$

*then the pair of the mappings* *T* *possesses a fixed point* *u*, *that is*, $Tu=u$.

#### Proof

It is sufficient to take $S=T$ in Theorem 2.1. □

#### Example 2.2

Let $X=[0,2]$ and define $d_{\theta}:X\times X\rightarrow [0,\infty )$ and $\theta:X\times X\rightarrow [1,\infty )$ by

$$ d_{\theta}(x,y)=\textstyle\begin{cases} (x-y)^{2} & \text{if }x,y \in [1,2 ],\\ \vert x-y \vert & \text{otherwise}, \end{cases} $$

respectively

$$ \theta(x,y)=\textstyle\begin{cases} x+y+1 & \text{if }x,y\in [1,2 ],\\ 1 & \text{otherwise.} \end{cases} $$

Let the self-map $T:X\rightarrow X$ be defined by

$$ T(x)=\textstyle\begin{cases} \frac{x}{8} & \text{ if } x \in[0,1)\\ \sqrt{-x^{2}+3x-2} & \text{ if } x \in[1,2]. \end{cases} $$

Define also $\alpha, \beta:X\times X\rightarrow [0,\infty )$ by

$$ \alpha(x,y)=\textstyle\begin{cases} 2 & \text{if } x,y \in[0,1],\\ 0 & \text{otherwise}, \end{cases} $$

and

$$ \beta(x,y)=\textstyle\begin{cases} 1 & \text{if } x,y \in[0,1],\\ 2 & \text{if } x=2, y=0,\\ 0 & \text{otherwise}. \end{cases} $$

We show that *T* is $(\alpha,\beta)$-orbital-cyclic admissible. Let $x,y\in X$ such that $\alpha(x,Tx)\geq1$ and $\beta(x,Tx)\geq1$. Then $x,y\in[0,1)$. On the other hand, if $x\in[0,1)$, then $Tx\leq1$ and $T^{2}x\leq1$. It follows that $\alpha(Tx,T^{2}x)\geq1$ and $\beta (Tx,T^{2}x)\geq1$. Thus, the assertion holds. For $x=0$, we have $T0=0$ and $\alpha (0, T0 )\geq1$, respectively, $\beta(0, T0)\geq1$, so assumption (ii) is satisfied. Let now $\{x_{n} \}$ be a sequence in *X* such that $x_{n}\rightarrow x$. Then $\{x_{n} \}\subset [0,1 ]$ and $x\in[0,1]$. This implies that $\alpha(x, Tx)\geq1$.

For $x_{0}\in[0,1)$, we get $T^{n}x_{0}=\frac{x_{0}}{8^{n}}$ and $\lim_{n,m\rightarrow\infty}\theta(T^{n}x_{0}, T^{m}x_{0})=1$. If $x_{0}\in[1,2]$, $Tx_{0}\leq\frac{1}{4}$, and $\lim_{n,m\rightarrow\infty}\theta(T^{n}x_{0}, T^{m}x_{0})=1$. So, assumption (i) is satisfied for $k=\frac{1}{3}$. We have the following cases:

(a): For $x,y\in[0,1)$, we get

$$\begin{aligned} &d_{\theta}(x,y)= \vert x-y \vert , \qquad d_{\theta}(Tx,Ty)= \frac{1}{8} \vert x-y \vert ,\\ & d_{\theta}(x,Tx)= \frac{7}{8}x,\qquad d_{\theta}(y,Ty)=\frac{7}{8}y. \end{aligned}$$

Replaced in (26) we get

$$ \alpha(x,Tx)\beta(y,Ty)d_{\theta}(Tx,Ty)\leq\frac{1}{3}\cdot \bigl[d_{\theta}(x,Tx)+d_{\theta}(y,Ty) \bigr] $$

or

$$ 2\frac{ \vert x-y \vert }{8}\leq\frac{1}{3} \biggl[\frac{7x}{8}+ \frac {7y}{8} \biggr]=\frac{7x+7y}{24}, $$

which is true for any $x,y\in[0,1)$.

(b): For $x=1$ and $y=2$, we know that $\alpha(1,T1)=\alpha(1,0)\geq 1$ and $\beta(T1, T^{2}1)=\beta(0,0)\geq1$, and $\beta(2, T2)=\beta (2,0)\geq1$ and $\alpha(T2, T^{2}2)=\alpha(0,0)\geq1$. But in this case (26) is obvious, because $d_{\theta}(T1,T2)=0$.

(c): For $x\in[0,1)$ and $y=2$, (26) becomes

$$ \alpha(x,Tx)\beta(2,T2)d_{\theta}(Tx,T2)\leq\frac{1}{3} \bigl[d_{\theta}(x,Tx)+d_{\theta}(2,T2) \bigr] $$

or

$$ \frac{x}{2}=4\cdot\frac{x}{8}\leq\frac{1}{3} \biggl[ \frac{7x}{8}+2 \biggr]=\frac{7x+16}{24}. $$

(d): For all other cases, $\alpha(x,Tx)=0$ or $\beta(x,Tx)=0$, and for this reason inequality (26) holds. Therefore, all the conditions of Corollary 2.1 are satisfied and *T* has a fixed point, $x=0$.

#### Corollary 2.2

*Let* *T* *be a self*-*mapping on a complete extended*-*bMS* $(X,d_{\theta})$ *such that* *T* *is an* *α*-*orbital admissible mapping*. *Suppose that*

(i) *for each* $x_{0}\in X$, $\lim_{n,m\rightarrow\infty}\theta (x_{n},x_{m})<\frac{1-k}{k}$, *where* $x_{n}=T^{n}x_{0}$, $n=1,2,\ldots $ ;
(ii) *there exists* $x_{0}\in X$ *such that* $\alpha (x_{0},Tx_{0})\geq1$;
(iii) *either* *T* *is continuous*, *or*
(iii*) *if* ${x_{n}}$ *is a sequence in* *X* *such that* $x_{n}\rightarrow u$, *then* $\alpha(u, Tu))\geq1$.

*Moreover*, *if for all* $x,y\in X$ *and* $k\in [0,\frac{1}{2} )$

27 $$ \alpha(x,Tx)\alpha(y,Ty)d_{\theta}(Tx,Ty)\leq k \bigl[d_{\theta}(x,Tx)+d_{\theta}(y,Ty) \bigr], $$

*then the pair of the mappings* *T* *possesses a fixed point* *u*, *that is*, $Tu=u$.

#### Proof

It is sufficient to take $\beta(x,y)=\alpha(x,y)$ in Corollary 2.1. □

#### Example 2.3

Let $X=[0,2]$ be endowed with extended *b*-metric $d_{\theta}:X\times X\rightarrow [0,\infty )$, defined by $d_{\theta}(x,y)=(x-y)^{2}$, where $\theta:X\times X\rightarrow [1,\infty )$, $\theta(x,y)=x+y+1$. Let $T:X\rightarrow X$ such that

$$Tx=\textstyle\begin{cases} \frac{x+1}{3} & \text{if }x \in [0,1 ],\\ \frac{x}{2} & \text{if }x \in(1,2]. \end{cases} $$

Define also $\alpha:X\times X\rightarrow (0,\infty )$ as

$$\alpha(x,y)=\textstyle\begin{cases} 1 & \text{if }(x,y) \in \{ [0,\frac{1}{2} ]\times [0,\frac{1}{2} ] \}\cup \{ [\frac{1}{2},1 ]\times [\frac{1}{2},1 ] \},\\ 0 & \text{otherwise}. \end{cases} $$

We prove that Corollary 2.2 can be applied to *T* for $k=\frac {1}{4}$, but Theorem 1.1 cannot be applied to *T*. We show that *T* is an *α*-orbital admissible mapping. If $x,y\in [0,\frac{1}{2} ]$, then $Tx\leq\frac{1}{2}$ and $T^{2}x\leq1$. Thus, $\alpha(x,Tx)\geq1$ implies $\alpha(Tx, T^{2}x)\geq1$. Similarly, we get that $\alpha(x,Tx)\geq1$ implies $\alpha(Tx, T^{2}x)\geq1$ for all $x,y\in [\frac{1}{2}, 1 ]$, so *T* is *α*-orbital admissible. In reason of the above arguments, $\alpha(0,T0)=\alpha(0, \frac {1}{3})\geq1$. Thus, assertion (ii) holds.

Note that, for each $x_{0}\in X$, $T^{n}x_{0}=\sum_{k=1}^{n} (\frac {1}{3} )^{n}+\frac{x_{0}}{3^{n}}$ and $\lim_{n\rightarrow\infty }T^{n}x_{0}=\frac{1}{2}$. Hence,

$$\lim_{n,m\rightarrow\infty}\theta \bigl(T^{n}(x_{0}), T^{m}(x_{0}) \bigr)=2\cdot \frac{1}{2}+1=2< 3= \frac{1-k}{k}. $$

So assumption (i) is satisfied, and because $\alpha (\frac{1}{2}, T\frac{1}{2} )=\alpha (\frac{1}{2},\frac{1}{2} )\geq1$, assumption (iii*) is also satisfied. Let $x,y\in [0,\frac{1}{2} ]$, or $x,y\in [\frac {1}{2},1 ]$. We have

$$ d(Tx,Ty)=\alpha(x,Tx)\alpha(y,Ty)d(Tx,Ty)=\frac{ (x-y )^{2}}{9} $$

and

$$ k\cdot \bigl[d_{\theta}(x,Tx)+d_{\theta}(y,Ty) \bigr]= \frac{1}{4} \biggl[\frac {(2x-1)^{2}}{9}+\frac{(2y-1)^{2}}{9} \biggr]= \frac{4x^{2}+4y^{2}-4x-4y+2}{36}. $$

Replaced in inequality $(28)$, we get

$$ \frac{x^{2}-2xy+y^{2}}{9}\leq\frac{4x^{2}+4y^{2}-4x-4y+2}{36}, $$

or, equivalently,

$$ 8xy-4x-4y+2\geq0\quad \Leftrightarrow\quad(2x-1) (2y-1)\geq0. $$

Hence, inequality $(28)$ is satisfied. In other cases, inequality $(28)$ is obviously satisfied, because $\alpha(x,y)=0$. Therefore, all conditions of Corollary 2.2 are satisfied and *T* has a unique fixed point, $x=\frac{1}{2}$.

Let $x=2$ and $y=3$. Then

$$ d_{\theta}(T2,T3)=d_{\theta}(9,16)=25>k=k\cdot d_{\theta}(2,3) $$

for any $k<1$.

### Uniqueness

Notice that in this section we investigate the existence of (common) fixed points of certain operators. For the uniqueness of a fixed point of the observed results, we will consider the following hypothesis.

(H) For all $x,y\in\operatorname{CFix}(T)$, we have $\alpha(x,Tx)\geq1$ and $\beta(y,Sy)\geq1$.

Here, $\operatorname{CFix}(T)$ denotes the set of common fixed points of *T* and *S*.

#### Theorem 2.2

*Adding condition* (H) *to the hypotheses of Theorem *2.1, *we obtain that* *u* *is the unique fixed point of* *T*.

#### Proof

Suppose, on the contrary, that *v* is another fixed point of *T*. From (H), there exists $v\in X$ such that

28 $$ \alpha(u,Tu)\geq1\quad \text{and}\quad \beta(v,Sv)\geq1. $$

Since *T* satisfies (9), we get that

$$ d_{\theta}(Tu,Sv)\leq\alpha(u,Tu)\beta(v,Sv)d_{\theta}(Tu,Sv)\leq k \bigl[d_{\theta}(u,Tu)+d_{\theta}(v,Sv) \bigr], $$

which yields that

$$d_{\theta}(u,v) \leq0. $$

Since the inequality above is possible only if $d_{\theta}(u,v)=0$, that is, $u=v$. This is a contradiction. Thus we proved that *u* is the unique fixed point of *T*. □

Notice also that instead of hypothesis (H), one can suggest different conditions, see, e.g., [15].

## Conclusions

It is clear that one can list several consequences from our results. By letting $\theta(x,y)=s$, constant, with $1\leq s <\frac{k-1}{k}$ in Theorem 2.1 (analogously, in Corollary 2.1 and Corollary 2.2), we get corresponding fixed point results in the setting of standard *b*-metric space.

On the other hand, regarding the techniques used in [15], one can derive another set of corollaries, by choosing the admissible mapping in a proper way. In this way, for example, several existing fixed point results in the literature in the setting of partially ordered metric spaces can be derived. Furthermore, the analogs of fixed point results for cyclic contractions can be found.
